# Analysis of Two Infrared Bands of CH_2_D_2_*

**DOI:** 10.6028/jres.067A.004

**Published:** 1963-02-01

**Authors:** Wm. Bruce Olson, Harry C. Allen, Earle K. Plyler

## Abstract

Two infrared absorption bands of CH_2_D_2_ have been analyzed in the semirigid rotor approximation. These are the A-type band at 2671.67 cm^−1^ and the C-type band at 4425.61 cm^−1^. The A-type band has previously been assigned as *v*_3_+*v*_9_, and the C-type band is tentatively assigned as *v*_3_+*v*_6_ The upper state of the A-type band is perturbed presumably by the close lying level 2*v*_5_. This interaction has not been investigated. The following values were found for the rotational constants of the ground vibrational state: *A*_0_=4.303 cm^−1^, *B*_0_= 3.504 cm^−1^, *C*_0_= 3.049 cm^−1^.

## 1. Introduction

The rotational-vibrational spectra of all the deuterated species of methane except CH_2_D_2_ have been well investigated [1].^1^

Methane and CD_4_ are spherical tops while CH_3_D and CD_3_H are symmetric rotors; on the other hand CH_2_D_2_ is an asymmetric rotor. It seemed of interest to determine the rotational constants of CH_2_D_2_ in order to have a set of constants for each of the species. Fortunately, high resolution spectra could be obtained for both an A-type and a C-type band for this molecule. As recently discussed [2], this is sufficient data to enable a good determination of the ground state constants using the complementary $\Delta F_{2}^{\prime \prime }$ values obtained from the two bands. These two bands have been analyzed in the semirigid approximation to yield good values of the ground state constants. Unfortunately a perturbation in the excited state of the A-type band introduces an uncertainty in the effective constants for this band that is larger than can be justified for the precision of the data. Only transitions involving levels with low values of the rotational quantum numbers have been used in the analysis in order to minimize the effect of centrifugal distortion.

## 2. Experimental Procedure

The spectra were recorded with the grating instrument of the Infrared Spectroscopy Section [3] using a 10,000 lines/in. grating with a ruled area of about 6×8 in. A cooled PbS photoconductive cell was used as the detector.

Both the A- and C-type bands were recorded using a multiple reflection cell with a total path length of 6 m, filled with CH_2_D_2_ to a pressure of 2 cm of Hg. The C-type band was observed in the second order of the grating and the A-type band in the first. The CH_2_D_2_ obtained from Merck & Co., Ltd., had a stated minimum purity of 98%.

The wavelengths of the lines were measured using higher order infrared emission lines of the rare gases as standards, and interpolating between them through the use of the fringes of a Fabry-Perot interferometer as previously described [4].

## 3. Theory

Preliminary to the actual analysis of the spectra the mean square values of the angular momenta about the three principal axes of inertia and the intensities were calculated.

Assuming tetrahedral geometry and identical bond lengths of 1.094 A for CH_2_D_2_ the moments of inertia, reciprocal moments in cm^−1^ units, and the asymmetry parameter *κ* were obtained.

For the calculated value of *κ*=−0.27, *α*, *β*, and *γ*, as defined by Allen [2], were obtained by linear interpolation in published tables [5] of *E*(*κ*) for each energy level.

*α*, *β*, and *γ* may be shown to be identical with $\langle P_{z}^{2}\rangle$, $\langle P_{x}^{2}\rangle$, and $\langle P_{y}^{2}\rangle$, respectively, in an *I^r^* representation [6]. The energy of a rotational level in a given vibrational state, neglecting centrifugal distortion, may be written as [2]:

$$E\left(J_{K_{-1,}K_{1}}\right)=\alpha A_{v}+\beta B_{v}+\gamma C_{v},$$

and the difference between two rotational levels in the same vibrational state as:

$$\Delta F=\Delta \alpha A_{v}+\Delta \beta B_{v}+\Delta \gamma C_{v}.$$

The Δ*α’*s, Δ*β*’s and Δ*γ*’s for each of the Δ*F*_2_’s observable from strong transitions in the A- and C-type band were calculated.

The strong transitions for the A-type band are those which satisfy the selection rules:

$$\Delta J=0,\;\pm 1\;\Delta K_{-1}=0\;\Delta K_{1}=\pm 1,$$

and for the C-type band the strong transitions are those for which

$$\Delta J=0,\;\pm 1\;\Delta K_{-1}=\pm 1\;\Delta K_{1}=0.$$

The relative intensities of the transitions were calculated by combining the Boltzmann factor, calculated using the estimated moments of inertia, and nuclear spin statistics with the appropriate line strengths from published tables [7].

The nuclear spins of the equivalent pairs of hydrogen and deuterium atoms in CH_2_D_2_ give rise to degeneracies of the rotational levels. For the ground vibrational state the statistical weight factors are 15 for the symmetric (A) rotational levels and 21 for the antisymmetric (B) levels.

The A-type band at 2672 cm^−1^ has been previously observed by Wilmshurst and Bernstein [8] who assigned it to the combination *v*_3_+*v*_9_. As the observed band type is consistent with this assignment and there should be no other bands of this type near 2670 cm^−1^, there appears to be no reason to doubt the assignment.

The C-type band at 4425 cm^−1^ can best be assigned at *v*_3_+*v*_6_ as this seems to be the only combination which would give a C-type band in the region 4400–4500 cm^−1^.

## 4. Analysis

As each of the bands was isolated from others of comparable intensity, initial assignments, with the aid of the calculated intensities, could be made by inspection. From the initial assignments Δ*F*_2_ values were obtained, and with the calculated Δ*α’*s, Δ*β*’s and Δ*γ*’s were used to refine the values of *A*, *B*, and *C* in the ground and upper vibrational states. From these values of *A*, *B*, and *C*, *α*’s, *β*’s, and *γ*’s pertinent to the value of *κ* in each vibrational state were calculated as before.

A trial spectrum was then calculated from the expression:

$$v=v_{0}+\alpha^{\prime }A_{v}+\beta^{\prime }B_{v}+\gamma^{\prime }C_{v}-\alpha A_{0}-\beta B_{0}-\gamma C_{0}.$$

From the trial spectrum and intensities more transitions could be assigned, enabling further refinement of the reciprocal moments of inertia.

While this iterative procedure worked well with the C-type band, Coriolis perturbations in the A-type band caused some difficulty in definitely locating some of the transitions.

The lowest observably perturbed level in the *v*_3_+*v*_9_ vibrational state is the 4_13_ level which is pushed down by 0.24 cm^−1^. For *J*=5, the levels 5_05_, 5_14_, and 5_23_ are all perturbed, and for *J*= 6 over half of the levels are perturbed.

This perturbation has not been investigated in detail. It probably arises through interaction with the vibrational state 2*v*_5_, the fundamental of which is theoretically inactive in the infrared, but appears to have been observed at 1329 cm^−1^ [8], the transitions becoming allowed through Coriolis perturbation. *v*_5_ has apparently been observed in the Raman also at 1333 cm^−1^ [9].

No account was taken of the effect of centrifugal distortion in this analysis. Since only levels with low *J* values were used in the analysis, the effect of this correction on the rotational constants was minimized. No systematic differences between the observed and calculated spectra were noticed until rather high *J* values were reached. In these regions of the absorption serious overlapping of transitions make the unique assignment of transitions to observed absorption peaks doubtful.

Although no statistical analysis of the data was made, the excellent agreement between the observed and calculated Δ*F*_2_ values for the ground state, and the sensitivity of the calculated Δ*F*_2_ values to values of the rotational constants seem to indicate a probable error of the order of ±0.002 cm^−1^ for each of the ground state constants. The agreement between the calculated and observed values of Δ*F*_2_ for the ground state may be seen in table 1.

The constants for the excited states of these bands cannot be determined as precisely as those for the ground state with the available Δ*F*_2_ values, but ±0.005 cm^−1^ would seem to be a generous estimate of the probable error in the constants for these states. The calculated and observed Δ*F*_2_ values for the excited states are compared in tables 2 and 3.

The constants determined for the three vibrational levels are given in table 4. The band origins were determined from the best fit between the observed and calculated spectra for low *J* values.

## Figures and Tables

**Figure 1:**
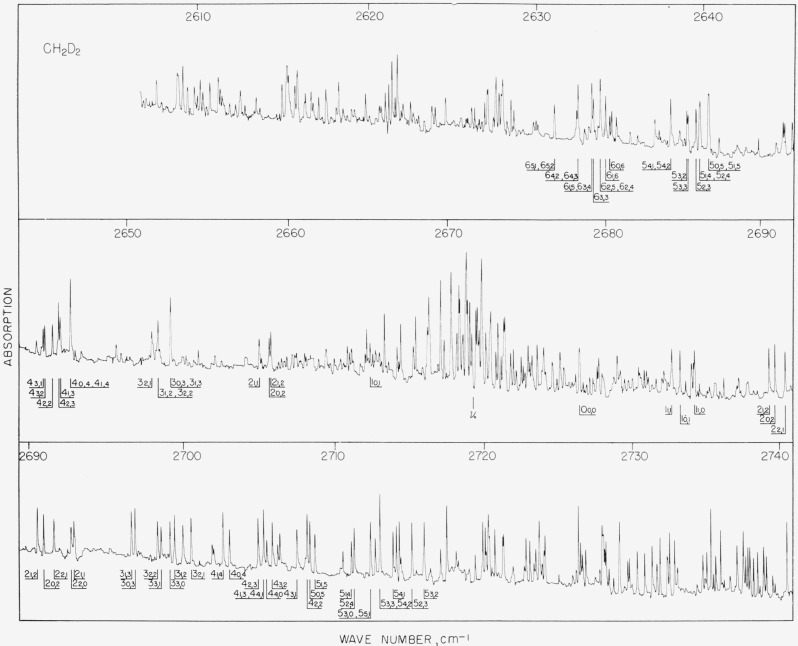
The A-type hand of *CH*_2_*D*_2_ at 2672 cm^−1^. The identification given is the ground state designation of $J_{K_{-1},\;K_{1}}$ for *R*_0,1_ transitions to the high wave number side of the band origin, and for *P*_0_, $\bar{1}$ transitions to the low wave number side of the band origin.

**Figure 2:**
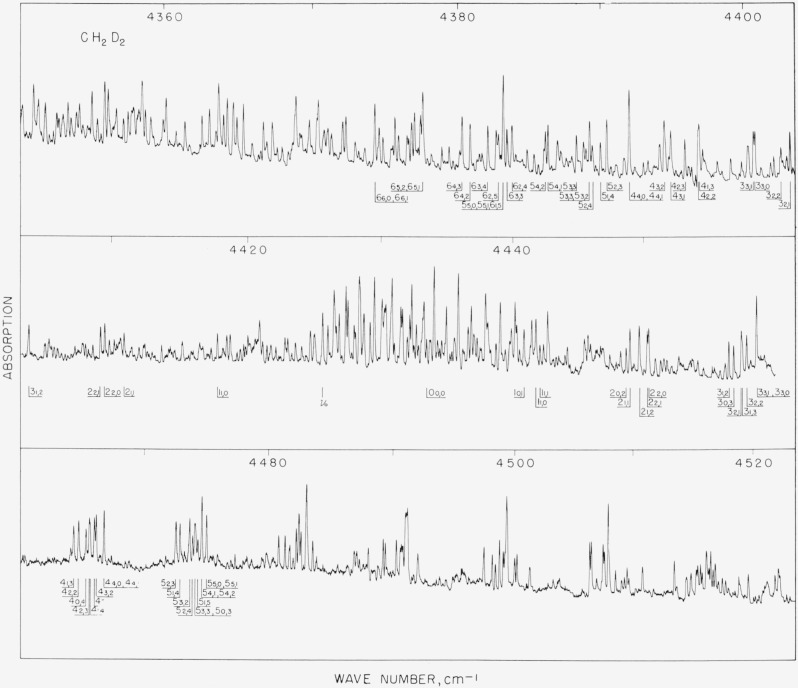
The C-type band of *CH*_2_*D*_2_ at 4426 cm^−1^ The identification given is the ground state designation of $J_{K_{-1},\;K_{1}}$ for *R*_1,0_ transitions to the high wave number side of the band origin, and for $P_{\bar{1}}$, 0 transitions to the low wave number side of the band origin.

**Table 1:** Ground state $\Delta F_{2}^{\prime \prime }$

A-type band			C-type band		
$\Delta F_{2}^{\prime \prime }$	Calculated	Observed	$\Delta F_{2}^{\prime \prime }$	Calculated	Observed
					
	*cm*^−1^	*cm*^−1^		*cm*^−1^	*cm*^−1^
0_00–_2_02_	19.513	19.55	0_00–_2_20_	23.911	23.92
1_01_–3_03_	32.103	32.10	1_01_–3_21_	37.524	37.53
1_11_–3_13_	31.547	31.55	1_11_–3_31_	41.285	41.29
1_10_–3_12_	33.791	33.79	1_10_–3_30_	40.862	40.88
2_02_–4_04_	44.373	44.38	2_02_–4_22_	51.644	51.64
2_12_–4_14_	43.976	43.98	2_12_–4_32_	55.068	55.06
2_11_–4_13_	46.958	46.96	2_11_–4_31_	53.908	53.91
2_21_–4_23_	45.754	45.76	2_21_–4_41_	58.305	58.27
2_20_–4_22_	47.277	47.25	2_20_–4_40_	58.165	58.16
3_03_–5_05_	56.501	56.50	3_03_–5_23_	66.378	66.37
3_13_–5_15_	56.300	56.28	3_13_–5_33_	69.201	69.20
3_12_–5_14_	59.715	59.69	3_12_–5_32_	67.212	69.23
3_22_–5_24_	58.539	58.54	3_22_–5_42_	71.733	71.73
3_21_–5_23_	60.967	60.96	3_21_–5_41_	71.127	71.12
3_31_–5_33_	59.463	59.47	3_31_–5_51_	75.463	75.43
3_30_–5_32_	60.141	60.13	3_30_–5_50_	75.431	75.43
4_04_–6_06_	68.637	68.61	4_04_–6_24_	81.651	81.64
4_14_–6_16_	68.559	68.53	4_14_–6_34_	83.676	83.63
4_13_–6_15_	72.042	71.98	4_13_–6_33_	81.042	81.03
4_23_–6_25_	71.147	71.11	4_23_–6_43_	85.399	85.38
4_22_–6_24_	74.350	74.33	4_22_–6_42_	83.950	83.93
4_32_–6_34_	72.585	72.55	4_32_–6_52_	88.728	88.65
4_31_–6_33_	74.092	74.07	4_31_–6_51_	88.532	88.52
4_41_–6_43_	72.848	72.81	4_41_–6_61_	92.665	92.56
4_40_–6_42_	73.062	73.02	4_40_–6_60_	92.660	92.56

**Table 2:** C-type band $\Delta F_{2}^{\prime }$ *A*=4.255 cm^−1^ *B*=3.590 cm^−1^ *C*=3.151 cm^−1^

Δ*F*′_2_	Calc	Obs
		
	*cm*^−1^	*cm*^−1^
0_00_–2_20_	23.918	23.96
1_01_–3_21_	37.927	37.94
1_11_–3_31_	41.086	41.12
1_10_–3_30_	40.685	40.70
2_02_–4_22_	52.436	52.44
2_12_–4_32_	55.238	55.25
2_11_–4_31_	54.163	54.18
2_21_–4_41_	57.925	57.91
2_20_–4_40_	57.778	57.80
3_03_–5_23_	67.521	67.51
3_13_–5_33_	69.726	69.74
3_12_–5_32_	67.944	67.93
3_22_–5_42_	71.753	71.74
3_21_–5_41_	71.131	71.15
3_31_–5_51_	74.891	74.82
3_30_–5_50_	74.854	74.82
4_04_–6_24_	83.064	82.95
4_14_–6_34_	84.534	84.48
4_13_–6_33_	82.268	82.15
4_23_–6_43_	85.784	85.82
4_22_–6_42_	84.431	84.42
4_32_–6_52_	88.552	88.51
4_31_–6_51_	88.324	88.32
4_41_–0_61_	91.899	91.83
4_40_–6_60_	91.890	91.83

**Table 3:** A-type band $\Delta F_{2}^{\prime }$ *A*=4.254 cm^−1^ *B*=3.654 cm^−1^ *C*=3.019 cm^−1^

$\Delta F_{2}^{\prime }$	Calc	Obs
		
	*cm*^−1^	*cm*^−1^
0_00_–2_02_	19.715	19.70
1_01_–3_03_	33.132	32.11
1_11_–3_13_	31.617	31.62
1_10_–3_12_	34.688	34.68
2_02_–4_04_	44.163	44.17
2_12_–4_14_	43.916	43.91
2_11_–4_13_^a^	47.748	47.51
2_21_–4_23_	46.516	46.54
2_20_–4_22_	48.979	48.96
3_03_–5_05_^a^	56.159	56.51
3_13_–5_15_	56.080	56.07
3_12_–5_14_^a^	60.078	59.35
3_22_–5_24_	59.144	59.11
3_21_–5_23_^a^	62.809	62.70
3_31_–5_33_	60.862	60.86
3_30_–5_32_	62.271	62.59

a Perturbed levels.

**Table 4:** Rotational and vibrational constants

	Ground state	*v*_3_+*v*_9_	*v*_3_+*v*_6_
			
	*cm*^−1^	*cm*^−1^	*cm*^−1^
*A*	4.303	4.254	4.255
*B*	3.504	3.654	3.590
*C*	3.049	3.019	3.151
*v*_0_		2671.67	4425.61

## Footnotes

1 Figures in brackets indicate the literature references at the end of this paper.

## References

[1] Hecht KT (1960). J Mol Spectroscopy.

[2] Allen HC (1961). Phil Trans Roy Soc London.

[3] Gailar N, Plyler EK (1955). J Research NBS.

[b4-jresv67an1p27_a1b] 4E. K. Plyler, L. R. Blaine, and E. D. Tidwell, ibid., **55**, 279 (1955).

[5] Townes CH, Schawlow AL (1955). Microwave Spectroscopy.

[6] Bragg JK, Golden S (1949). Phys Rev.

[7] Cross PC, Hainer RM, King GW (1944). J Chem Phys.

[8] Wilmshurst JK, Bernstein HJ (1957). Can J Chem.

[9] Mac Wood GE, Urey HC (1936). J Chem Phys.

